# A Novel Trajectory Feature-Boosting Network for Trajectory Prediction

**DOI:** 10.3390/e25071100

**Published:** 2023-07-23

**Authors:** Qingjian Ni, Wenqiang Peng, Yuntian Zhu, Ruotian Ye

**Affiliations:** School of Computer Science and Engineering, Southeast University, Nanjing 211189, China

**Keywords:** trajectory prediction, feature boosting, goal-driven

## Abstract

Trajectory prediction is an essential task in many applications, including autonomous driving, robotics, and surveillance systems. In this paper, we propose a novel trajectory prediction network, called TFBNet (trajectory feature-boosting network), that utilizes trajectory feature boosting to enhance prediction accuracy. TFBNet operates by mapping the original trajectory data to a high-dimensional space, analyzing the change rules of the trajectory in this space, and finally aggregating the trajectory goals to generate the final trajectory. Our approach presents a new perspective on trajectory prediction. We evaluate TFBNet on five real-world datasets and compare it to state-of-the-art methods. Our results demonstrate that TFBNet achieves significant improvements in the ADE (average displacement error) and FDE (final displacement error) indicators, with increases of 46% and 52%, respectively. These results validate the effectiveness of our proposed approach and its potential to improve the performance of trajectory prediction models in various applications.

## 1. Introduction

Trajectory prediction is a crucial technology with practical applications in various fields, including automatic driving [1], robotics [2], and surveillance systems [3]. In autonomous driving systems, accurate prediction of the trajectories of surrounding vehicles, pedestrians, and other traffic participants is crucial for safety and decision-making. In robot navigation and path planning, predicting the trajectories of objects in the surrounding environment helps robots avoid obstacles, prevent collisions, and optimize path selection. In security surveillance, trajectory prediction assists security systems in analyzing and identifying suspicious behavior or abnormal movements, used in areas such as video monitoring, intrusion detection, and behavior analysis. In traffic management, by predicting the motion trajectories of vehicles, pedestrians, and bicycles, traffic authorities can better plan roads, traffic signals, and traffic flow to improve efficiency and reduce congestion. Its ability to predict and prevent traffic accidents, improve traffic flow management, and enhance logistics and distribution efficiency makes it an essential tool in our daily lives.

In the context of traffic management, trajectory prediction can help us forecast traffic congestion and prevent accidents, improving road planning and traffic flow management. Additionally, in the navigation of autonomous vehicles and robots, trajectory prediction can enhance safety by predicting the trajectories of pedestrians and other vehicles, and by providing better path planning for making safe and efficient driving decisions. Moreover, in monitoring systems, trajectory prediction can be used to forecast the movement of people or vehicles in crowded scenes, improving the efficiency of tracking and monitoring systems. Finally, in logistics and distribution, trajectory prediction can optimize distribution efficiency, forecast the movement of distribution vehicles, and provide better support for distribution route planning.

However, trajectory prediction is a challenging research topic. The motion mode of an object is complex and uncertain and will be affected by various factors, such as obstacles, interactions with other objects, and the uncertain environment. These factors make the prediction of future motion nonlinear and complex, making it difficult to accurately forecast the trajectory of an object. Additionally, the future movement of an object also depends on its goals, plans, and intentions, which are difficult to model and predict. As a result, developing accurate and efficient trajectory prediction methods remains an ongoing research challenge.

Early work on trajectory prediction primarily relied on Markov process models [4,5] and social force models [6,7]. However, with the rapid development of deep learning in recent years, researchers have started using deep neural networks to tackle trajectory prediction problems. AMGB [8] handles trajectory prediction in dynamic environments from a new perspective of predicting vehicle movement direction and distance. Zhang [9] proposed a dual-branch spatial-temporal graph neural network to automatically model and group view regions. Hui [10] proposed a trajectory prediction model based on a deep encoder–Cdecoder and a deep neural network (DNN), which introduces an attention mechanism into the traditional encoder–Cdecoder framework. Shi [11] proposed an integrated deep-learning-based two-dimensional trajectory prediction model that can predict combined behaviors. Recurrent neural networks (RNN), including long short-term memory (LSTM) and gated recurrent unit (GRU), have been used to model trajectory prediction [12,13,14,15,16,17,18]. These models can handle continuous data and consider the object’s past movements while making predictions.

Social-LSTM [13] is an architecture that connects LSTMs of different sequences and proposes a social pooling layer to handle different agent interactions. However, in many scenarios, multiple objects interact with each other, making it challenging to model uncertainty, unpredictability, and the intentions of different agents. Simple recurrent neural networks may not handle these interactions effectively. In recent years, graph neural networks (GNN) have gained popularity and achieved good results in many fields. GNNs are designed to handle interactions between different nodes. As a result, researchers have applied GNNs to trajectory prediction [19,20,21,22,23,24,25]. The primary function of GNNs is to model the interactions between different nodes, which can help in dealing with the interaction between multiple objects, such as vehicles, pedestrians, and obstacles, in the case of autonomous vehicles. GNNs have shown great potential to improve the performance of trajectory prediction models.

Recent research has focused on generating multiple possible future trajectories because the future motion of objects is uncertain, and this approach may provide more realistic predictions and improve the safety of autonomous systems. For instance, a car may turn left or go straight at an intersection, leading to different future trajectories depending on the driver’s intended destination. Similarly, a person may produce multiple possible future trajectories depending on their intended destination. At present, multi-modal trajectory prediction is mainly based on generation methods, which can be divided into two categories: GAN generation methods [26,27,28] and conditional variational auto-encoder (CVAE) methods [16,21,29,30,31,32,33].

The proposed trajectory prediction network in this paper, called TFBNet, aims to improve the accuracy of trajectory prediction by using trajectory feature boosting. This approach maps the original trajectory data to a higher-dimensional space where the motion intention of the object can be mined. TFBNet consists of four main modules: (1) a trajectory feature-boosting module, which maps the trajectory data to a higher dimension and learns the distribution of future trajectories through a CVAE module; (2) a multi-recurrent goal mining module, which uses GRU to mine trajectory data in the high-dimensional space and form multiple prediction goals; (3) a goal feedback module, which links the prediction goals with the module and feeds back the prediction results to dynamically adjust the model and has the function of memory backtracking. It feeds back the historical trajectory prediction results to the subsequent prediction, which is convenient for the model to capture similar trajectories and assist in prediction; (4) a target aggregation module, which evaluates multiple predicted goals, selects effective goals, reduces high-dimensional trajectory data to low-dimensional, and generates the final predicted trajectory.

Understanding the intention of object motion is crucial to improving the accuracy of multi-modal trajectory prediction. Different intentions may produce completely different results, and effectively mining an object’s potential intention based on past trajectory observations is, therefore, essential. The key to improve the accuracy of multi-modal trajectory prediction is to determine the object’s intention accurately. In order to better capture the motion intent of objects, this paper maps the original trajectory data into a high-dimensional space and explores the motion intent of objects in that space.

The main contributions of this paper are as follows:

- A novel approach for trajectory prediction is proposed, which maps the original trajectory data to a high-dimensional space to better mine the object’s motion intention. The experimental results demonstrate that this approach can improve the accuracy of trajectory prediction by capturing the change law of the object’s motion trajectory more effectively.
- A dilated attention gating structure (DAGConv) is introduced and applied to both the CVAE module and the goal aggregation module. The results show that DAGConv can effectively extract useful information and significantly enhance the accuracy of trajectory prediction.
- A goal feedback structure is designed, which not only provides real-time feedback to the model, but also evaluates the output results of the trajectory representation module.
- A goal aggregation module is developed, which integrates the attention mechanism and the dilated attention gating structure. This module can evaluate multiple prediction goals, select effective ones adaptively, and generate the final predicted trajectory.

In summary, we propose a novel trajectory prediction model that exhibits improved predictive performance compared to other trajectory prediction models. Additionally, we provide a new approach for trajectory prediction by mapping the data into a high-dimensional space to better explore trajectory features.

The organizational structure of this paper is as follows: Section 1 provides an introduction; Section 2 introduces the relevant methods; Section 3 presents the model proposed in this paper to address the trajectory prediction problem and provides a detailed description of the model; Section 4 compares and analyzes the experimental results; Section 5 concludes the paper with a summary of the key findings.

## 2. Related Work

Trajectory prediction involves predicting the future motion trajectory of an object based on its past motion trajectory. Accurate prediction of an object’s future motion trajectory would greatly benefit applications such as autonomous driving and robotics. In trajectory prediction, better exploration and understanding of an object’s motion intent are key to improving the performance of trajectory prediction. In recent years, trajectory prediction has made a lot of progress. In order to enable agents to interact better, Agentformer (agent-aware transformers for socio-temporal multi-agent forecasting) [33] is proposed. Based on the Transformer architecture, Agentformer has designed an agent-aware attention structure, which allows the model to distinguish different agents, thus improving the ability to interact with information. Social-GAN (socially acceptable trajectories with generative adversarial networks) [26] improves on Social-LSTM, focusing on generating trajectories that conform to social norms. At the same time, the social LSTM model is pooled every step into only one pool in the known trajectory change stage, reducing the computational overhead and improving the prediction speed of the model. In order to better deal with the interaction between agents, a graph neural network is undoubtedly a good choice [19,20,21,22,23,24,25], Social-bigat (social bicycle-gan and graph attention networks) [19] combines GAT (graph attention network) and GAN (generative adversarial networks). This method also takes into account the relationship between noise and prediction trajectory, and proposes a latent encoder, which links the prediction trajectory with noise to improve the accuracy of the prediction trajectory. In contrast to the model with a cyclic recursive structure, Social-STGCNN (social spatio-temporal graph convolutional neural network) [20] uses a TCN (temporary revolutionary network) to replace the cyclic recursive structure. Compared with Social-bigat, Social-STGCNN improves the prediction accuracy with fewer parameters and better training speed. STAR (spatio-temporal graph transformer networks) [34] is different from the graph convolution network. STAR takes the agent dimension as the time dimension of the transformer and uses the transformer structure to realize the interaction between agents. RSBG (recursive social behavior graph) [22] proposes a social behavior graph and uses a recursive structure to mine interactive information. Although the graph neural network can extract the interaction well, it is more suitable for the prediction of fixed nodes, such as traffic flow prediction. The node of traffic flow prediction is the sensor fixed on the road, and its position is unchanged. However, for trajectory prediction, the scene is likely to change constantly. The number of agents in different scenes is generally different, and the number of agents at different times in the same scene may also be different. However, to construct a graph structure, the number of nodes of the graph needs to be determined in advance. Therefore, for trajectory prediction, the graph neural network cannot play its role well. The semantic information of the trajectory context also has an impact on prediction. CSCnet (contextual semantic consistency network) [35] considers the semantic consistency of the context of trajectory prediction in a congested space to improve the prediction accuracy. Ynet (a scene compliant trajectory forecasting network) [36] uses multimodal input to input a semantic map, RGB map and other information into the model. However, this requires large datasets. Some datasets do not have such data, and the adaptability is not so good. Use of a physical model is also one of the trajectory prediction methods. It is also one of the feasible methods to integrate a physical model into a deep learning model. NSP-SFM (neural social physics models–social force models) [37] combines a physical model with a deep learning model, uses a physical model with learnable parameters, and obtains good prediction results. For multi-modal trajectory prediction, use of a goal-driven method has become one of the research hotspots in recent years [29,30,31,38,39,40]. Tnt (target-driven trajectory prediction) [38] is guided by the endpoint of the trajectory and predicts the trajectory in three stages. First, the final goal is generated, and then the trajectory is generated based on these goals. Finally, these trajectories are filtered and scored to obtain the prediction results. PECNet (predicted endpoint conditioned network) [31] is also used to generate the final goal point first, and then uses the goal point to assist in prediction, while paying attention to the generation of trajectories that conform to social norms. Bitrap (bi-directional pedestrian trajectory prediction) [29] uses a bidirectional loop decoder based on the goal condition to estimate the endpoint of the trajectory and improve the long-term prediction ability of the model. SGNet (stepwise goal-driven networks) [30] improves trajectory prediction accuracy by generating multiple goals. MemoNet (retrospective-memory-based network) [40] proposes a memory backtracking structure, which predicts the movement intention of the agent by looking for similar scenes in the training data. Each prediction can find similar scenes in the memory, and, at the same time, the current scene is also stored in the memory. The prediction process of MemoNet is also divided into two steps: first, predict the endpoint, and then generate the trajectory according to the end point.

In contrast to previous models, the model proposed in this paper proposes a new idea, which involves mapping the original trajectory data to a higher dimension to space, thus improving the representation of the trajectory features and facilitating better mining of trajectory data. In order to better mine the trajectory features, this paper proposes a dilated attention gating structure and a multi-recurrent goal aggregation structure to improve the ability of the model to mine trajectory features. At the same time, this paper considers the impact of the goal on the trajectory prediction, and designs a goal feedback structure to assist in the prediction.

## 3. Proposed Model

### 3.1. Problem Formulation

The trajectory prediction in this paper is based on the observed trajectory of the agent in the past to predict the trajectory of the agent in the future. Specifically, X = $\left(X_{t-\mathrm{obs}+1},\;X_{t-\mathrm{obs}+2},\;\ldots ,\;X_{t}\right)\in R^{B\times obs\times C}$ represents the trajectory data with the length of *obs* observed in the past, where *B* represents the batch size, and *C* represents the number of features entered, including the trajectory features (coordinates, speed, acceleration, etc.). $Y=\left\{y_{t+1},\;y_{t+2},\;\ldots ,\;y_{t+pred}\right\}\in R^{B\times pred\times pos}$ represents the prediction goal, where pred represents the predicted time length and pos represents the predicted coordinates. The prediction content in this paper is multimodal trajectory prediction, so the prediction result $Y_{\mathrm{pred}}=\left\{{\tilde{y}}_{t+1},\;{\tilde{y}}_{t+2},\;\ldots ,\;{\tilde{y}}_{t+\mathrm{pred}}\right\}\in R^{B\times \mathrm{pred}\times K\times \mathrm{pos}}$, where *K* represents the number of samples output.

### 3.2. The Architecture of TFBNet

**Figure 1:**
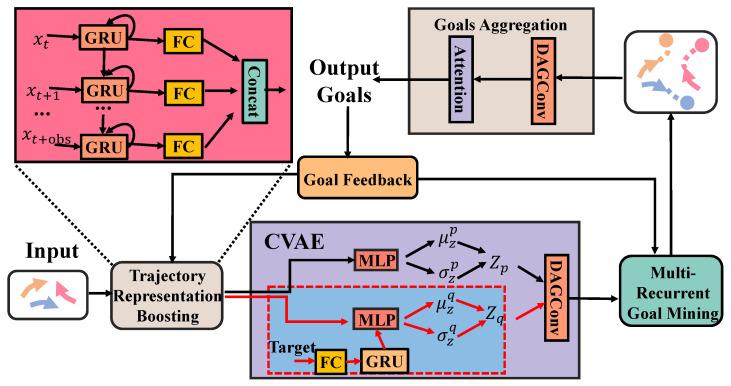
Model architecture of the proposed TFBNet.

Figure 1 depicts the overall architecture of TFBNet. The main idea behind TFBNet is to map the original trajectory data to a high-dimensional space, extract the trajectory features in that space, generate multiple goal trajectories, and finally use the goal aggregation module to combine them and reduce the high-dimensional data to a lower-dimensional space. TFBNet comprises four key components: a trajectory feature-boosting module, a multi-recurrent goal mining module, a goal aggregation module, and a goal feedback module. The trajectory feature-boosting module improves the representation of the original trajectory data, while the multi-recurrent goal mining module is responsible for extracting the trajectory features. The goal aggregation module is used to reduce the high-dimensional data to a low-dimensional space and to generate the final prediction results. Additionally, this paper introduces a dilated attention gating convolution (DAGConv), which is a crucial component of the CVAE and the goal aggregation module. The experimental results obtained demonstrate that DAGConv significantly improves the prediction accuracy of the model.

### 3.3. Trajectory Feature Boosting

#### 3.3.1. Trajectory Representation Boosting

To map the low-dimensional trajectory data to the high-dimensional space, this paper processes the data of each time step separately. In particular, multiple GRU structures are utilized to process the trajectory data of each time step and these structures are linked together according to the time relationship. This stacking method helps to mine the time dependence of the trajectory data and provides a more comprehensive representation of the trajectory. By processing each time step independently, the model is able to capture the information of not only the current time step, but also of the other time steps.

Furthermore, each GRU passes through an independent full connection layer, allowing for more information to be extracted from each time step. Finally, the information of all time steps is aggregated to obtain a more comprehensive representation of the trajectory data. By employing this method, the model is able to capture the complex and dynamic nature of the trajectory data and provide more accurate trajectory predictions. The formula is as follows:

(1) $$r_{t}=W_{r}\left(GRU\left(\mathrm{Dropout}\;\left(\mathrm{Concat}\;\left(x_{t},g_{r}\right)\right)\right)+b_{r}\right)$$

(2) $$r_{out}=\mathrm{Concat}\left(r_{t},r_{t+1},\ldots ,r_{t+obs}\right)$$

where, $x_{t}$ represents the original input at time *t*, $g_{r}$ represents the variable output from the goal feedback mechanism to the trajectory representing the lifting module, $r_{out}$ represents the output of the representation boosting module, $W_{r}$, and $b_{r}$ represent the training parameters of the model. Dropout refers to randomly dropping out some neuron connections, while Concat represents concatenation.

#### 3.3.2. Dilated Attention Gating

**Figure 2:**
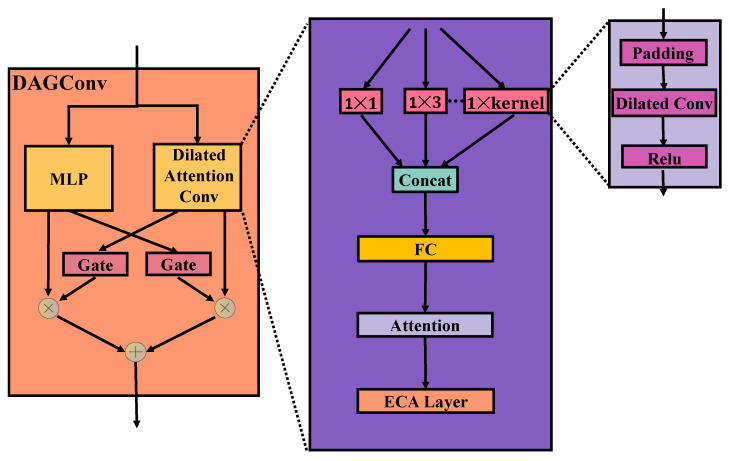
The architecture of DAGConv.

The structure of DAGConv (dilated attention gating convolution) is shown in Figure 2, which is mainly composed of DAConv (dilated attention convolution), MLP (multilayer perceptron), and a gating mechanism. DAConv and MLP separately mine the information and then each pass the gating, and, finally, the two results are added to obtain the final output. The following introduces the structure of DAConv:

As shown in Figure 2, DAConv uses multi-resolution expansion convolution to mine trajectory information at different levels. For the input data, the expansion convolution of different size convolution kernels is used to process the data separately, and then the results are organically combined through the attention mechanism. The attention mechanism can be introduced to adaptively select multi-resolution convolution results. It should be noted that the size of the convolution result is different, so this paper uses the boundary padding method to make the output result size the same. The expansion convolution increases the receptive field of the convolution kernel while keeping the number of parameters unchanged. Compared with RNN, it can greatly improve the training speed of the model. The attention formula is:

(3) $$\left.\alpha =W_{a}\cdot \sigma \left(W_{l}\left(\mathrm{input}\right)+b_{l}+\tilde{x}\right)\right)+b_{a}$$

(4) $$\gamma_{i}=\frac{\exp\left(\alpha_{i}\right)}{\sum_{j=1}^{n}\exp\left(\alpha_{j}\right)}$$

(5) $${Out}_{D}=\left[f\left(\gamma_{0}{\tilde{x}}_{0},\gamma_{1}{\tilde{x}}_{1},\ldots ,\gamma_{num}{\tilde{x}}_{num}\right)\right]$$

Among them, σ is the activation function *tanh*, input represents the initial input of DAGConv, and $\tilde{x}$ indicates the DAConv output. $W_{a}$, $W_{l}$, $b_{l}$, $b_{A}$ are trainable parameters of the model. ${Out}_{D}$ represents the output of the attention mechanism and *num* represents the number of different convolutions. In order to better select the trajectory goals and generate more accurate prediction results, this paper introduces a channel attention mechanism ECA layer [41] in DAConv. The ECA layer avoids dimensionality reduction and local cross-channel interaction, which improves the performance of the model while reducing the complexity of the model. The weight of channel $C^{i}$ is calculated as follows:

(6) $$\beta_{i}=\sigma \left(\sum_{j=1}^{k}w_{i}^{j}C_{i}^{j}\right),\;C_{i}^{j}\in \Psi_{i}^{k}$$

among $\Psi_{i}^{k}$ represents the set of *k* adjacent channels of $C^{i}$, $W_{k}\in R^{k\times d}$, *d* represents the number of channels, $W_{k}$ is set to:

(7) $$\left[\begin{array}{cccccccc} w^{1,1}\; & \cdots & w^{1,1} & 0 & 0 & \cdots & \cdots & 0\\ 0 & w^{2,2} & \cdots & w^{2,k+1} & 0 & \cdots & \cdots & 0\\ \vdots & \vdots & \vdots & \vdots & \ddots & \vdots & \vdots & \vdots \\ 0 & \cdots & 0 & 0 & \cdots & w^{d,d-k+1} & \cdots & w^{d,d} \end{array}\right]$$

The above *k* is a super parameter, which can be set manually or adaptively:

(8) $$k=f\left(d\right)={\left|\frac{\log_{2}}{\gamma }+\frac{b}{\gamma }\right|}_{odd}$$

Among them, γ is 2, and *b* is 1.

#### 3.3.3. CVAE

The CVAE module in this paper consists of three parts: a front network $p_{\theta }\left(Z|X_{t}\right)$, an identification network $q_{\varphi }\left(Z|X_{t},Y_{t}\right)$, and a generating network $p_{\omega }\left(c_{\mathrm{out}\;}|X_{t},Z\right)$. θ, φ, ω is the model parameter, $c_{out}$ is the output of the CVAE module. The mean value of the prior network generation is $\mu_{z}^{p}$, the variance is $\sigma_{z}^{p}$ distribution $N\left(\mu_{z}^{p},\sigma_{z}^{p}\right)$, the mean value of the recognition network generation is $\mu_{z}^{q}$, the variance is $\sigma_{z}^{q}$ distribution $N\left(\mu_{z}^{q},\sigma_{z}^{q}\right)$. Since identifying the network requires real future trajectory data $Y_{t}$, it can only be used in the training stage. When training, the model is distributed $N\left(\mu_{z}^{q},\sigma_{z}^{q}\right)$; when testing, the model is distributed $N\left(\mu_{z}^{p},\sigma_{z}^{p}\right)$. In particular, the DAGConv structure is introduced into the CVAE generation network. The experimental results show that DAGConv improves the performance of CVAE greatly.

### 3.4. Multi-Recurrent Goal Mining

The objective of this module is to thoroughly extract trajectory features and generate multiple trajectory sequences for different goals. Since an agent’s trajectory at a given time is influenced by its past trajectory and destination, the trajectory can be seen as a continuous sequence. Therefore, this paper introduces a multi-recurrent structure, which includes an attention mechanism, a forward GRU, and a backward GRU. Unlike the bidirectional GRU, we introduce the input of the goal feedback module. Additionally, our forward GRU and backward GRU are concatenated, and the input of the backward GRU is related to the output of the forward GRU. The forward GRU uses the attention mechanism to process the output of the goal feedback module and the pre-input. The output of the forward GRU is then fed into the backward GRU in reverse order of time. The backward GRU uses the output of the forward GRU to initialize its hidden layer state, and its final output is obtained by combining the backward GRU output with the output of the forward GRU. By incorporating this multi-recurrent structure, the model can more effectively capture the temporal dependencies in the trajectory data and generate multiple trajectory sequences for different goals.

(9) $$F_{\mathrm{out}}=GRU_{f}\left(\mathrm{Concat}\left(FC\left(h_{f}\right),\;\mathrm{Attention}\;\left(g_{m}\right)\right),h_{f}\right)$$

(10) $$\mathrm{Attention}\left(g_{m}\right)=g_{m}*\mathrm{Softmax}\left(W_{m}\left(\sigma \left(g_{m}\right)\right)+b_{m}\right)$$

(11) $$B_{\mathrm{out}\;}=GRU_{b}\left(\mathrm{Concat}\left(FC\left(h_{b}\right)\right),h_{b}\right)$$

(12) $$\begin{array}{cc} M_{\mathrm{out}\;} & =FC\left(\mathrm{Concat}\left(F_{\mathrm{out}},B_{\mathrm{out}}\right)\right) \end{array}$$

where, $GRU_{f}$ represents the forward GRU, $GRU_{b}$ represents the backward GRU, $F_{out}$ represents the output of the forward GRU, $B_{out}$ represents the output to the GRU, $g_{m}$ represents the output of the goal feedback module to this module, $h_{f}$ represents the hidden layer state of the forward GRU, $h_{b}$ represents the hidden layer state of the backward GRU, and $M_{out}$ indicates the output of this module.

### 3.5. Goals Aggregation

The module described above maps trajectory data to a high-dimensional space and generates multiple potential trajectories for the model to follow. Its function is to collect several candidate trajectories, map them back to a low-dimensional space, and produce the final prediction results. To accomplish this, the module combines the attention mechanism with DAGConv.

First, an adaptive method is employed to select the most suitable trajectories from among the many potential ones. Next, DAGConv is used to gather and merge these selected trajectories, eliminating any invalid ones and retaining only those that accurately represent the model’s intended goals. Finally, the resulting set of goal trajectories is used to generate the model’s predictions. The specific formula is:

(13) $$\begin{array}{cc} M_{att} & =M_{\mathrm{out}}*\mathrm{Softmax}\left(W_{\rho }\left(M_{\mathrm{out}}\right)+b_{\rho }\right) \end{array}$$

(14) $$\begin{array}{cc} Y_{pred} & =\;\mathrm{DAGConv}\;\left(M_{att}\right) \end{array}$$

### 3.6. Goal Feedback

The goal feedback module plays a crucial role in improving the trajectory prediction accuracy. By feeding back the generated trajectory goals to the model, the auxiliary model can better learn the trajectory sequence. Essentially, the goal feedback module functions as a memory backtracking module that feeds back the historical trajectory prediction results to aid in subsequent predictions. This feedback mechanism allows the model to capture similar trajectories and facilitates prediction.

As previously mentioned, the goal feedback module produces two outputs: one for the trajectory representation boosting module and another for the multi-recurrent goal mining module. However, the prediction goals generated by the model may contain errors that could accumulate over time and negatively impact prediction accuracy. To address this issue, this paper proposes an attention mechanism to filter out potentially problematic goals before feeding them back to the model. The attention mechanism works by evaluating the similarity between the predicted trajectory goals and the actual ground truth trajectories. Goals that deviate too far from the actual trajectory are deemed inaccurate and filtered out. This approach helps ensure that the feedback mechanism reinforces the model’s ability to predict accurate trajectories.

In summary, the goal feedback module is a vital component of the trajectory prediction model, facilitating improved learning and more accurate predictions. By incorporating an attention mechanism, the module can further enhance accuracy by filtering out inaccurate predictions and reinforcing accurate ones.

## 4. Experimental Results

### 4.1. Datasets

The dataset utilized in this paper is the publicly available ETH and UCY pedestrian trajectory dataset. These datasets are recorded from a third-person perspective and contain five subsets: ETH, HOTEL, UNIV, ZARA1, and ZARA2, featuring four unique scenes and a total of 1536 pedestrians. The dataset presents numerous challenging behaviors, such as couples walking together, group crossing, and formation and dispersion of groups. The data is collected at a frequency of 2.5 Hz, meaning that one frame of data is captured every 0.4 s.

To evaluate the model’s prediction performance, the paper adopts the leave-one-out method for data segmentation. This method involves using four of the datasets as training sets, while the remaining one is reserved for testing. For instance, to evaluate the model’s prediction performance for the HOTEL dataset, the ETH, UNIV, ZARA1, and ZARA2 subsets are utilized for training, while HOTEL is set aside for testing. This method ensures that the model is evaluated using all available data and that its predictive accuracy is robust across different datasets.

### 4.2. Experimental Settings

The experiments in this paper were all completed under the Linux server. The server configuration was CPU: Intel Xeon Gold 6226R × 2, GPU: NVIDIA TITAN RTX × 3.

This paper proposes that the hidden layer state of the GRU of the model is set to 512, the batch size is set to 128, and the training iteration is 50 times. The data of the past 8 time steps is used to predict the data of the next 12 time steps, and the number of samples is K = 20.

#### 4.2.1. Evaluation Metrics

In this paper, the average distance error (ADE) and the final distance error (FDE) are used as the evaluation indicators of the model. The smaller the value of these two evaluation indicators, the better the prediction performance of the model.

(15) $$\begin{array}{cc} ADE & =\frac{\sum_{t\in T_{\mathrm{pred}\;}}{∥\hat{y_{t}}-y_{t}∥}_{2}}{T_{\mathrm{pred}\;}} \end{array}$$

(16) $$\begin{array}{cc} FDE & ={∥{\hat{y}}_{t}-y_{t}∥}_{2},t=T_{\mathrm{pred}\;} \end{array}$$

where, $T_{pred}$ indicates the predicted goal time length, $\hat{y_{t}}$ represents the predicted value of the track at the time *t*, and $y_{t}$ is the true value of the track at time *t*.

#### 4.2.2. Baselines

This paper compares the proposed model with the following baselines:

1. **Social-LSTM** [13]: This model introduces a “social” pooling layer that allows LSTMs of spatially adjacent sequences to share their hidden states with each other.
2. **SGAN** [26]: This model combines sequence prediction and generating adversarial networks to predict trajectories.
3. **Sophie** [27]: This model utilizes two information sources, namely all path history and scene context information in the scene, and combines physical and social information using a social attention mechanism and physical attention.
4. **Social-bigat** [19]: This model is based on a graphical attention network, encoding reliable feature representations of social interactions between humans in the scene, and combining them with generative adversarial networks to generate multiple future trajectories.
5. **RSBG** [22]: A group-based social interaction model, which uses a graph convolution neural network to disseminate social interaction information in such a graph by recursively extracting social representation.
6. **MATF GAN** [42]: This model encodes the past trajectories and scene contexts of multiple agents into multi-agent tensors, and then applies convolutional fusion to capture multi-agent interactions while preserving the spatial structure and scene context of the agents.
7. **PSA-GRU** [17]: The model adopts a human social dual-attention network based on gated recursive units, fully utilizing important location nodes of personal historical trajectories and social information between pedestrians.
8. **Social-STGCNN** [20]: This model replaces aggregation methods by modeling interactions as graphs.
9. **CGNS** [43]: This model combines the advantages of conditional potential space learning and variable dispersity minimization, and uses an attention mechanism to utilize static context and interactive information.
10. **PIF** [44]: Adopting an end-to-end multitasking learning model that utilizes rich visual features about human behavior information and its interaction with the surrounding environment.
11. **NMMP** [28]: This model uses a separate branch to simulate the behavior of a single agent, an interaction branch to simulate the interaction between agents, and different wrappers to handle different input formats and features.
12. **FvTraj** [45]: This model is based on a multi-head attention mechanism and uses a social perception attention module to simulate social interaction between pedestrians, as well as a view perception attention module to capture the relationship between historical motion states and visual features.
13. **DSCMP** [46]: This model simulates dynamic interaction between agents by learning the spatial and temporal consistency of agents, as well as understanding the layout of contextual scenes. At the same time, a differentiable queue mechanism is designed, which can clarify the correlation between memory and learning long trajectories.
14. **STGAT** [23]: This model is based on a sequence-to-sequence architecture to predict the future trajectory of pedestrians. In addition to the spatial interaction captured by the graph attention mechanism at each time step, additional LSTM is also used to encode the temporal correlation of the interaction.
15. **TPNet** [47]: This model is divided into two stages to predict trajectories: first creating some suggested target trajectories, and then classifying and refining these trajectories to obtain the final predicted trajectory.

### 4.3. Experiment Results and Analysis

Table 1 presents the comparative experimental results of the proposed TFBNet and the baseline model for predicting the next 12 time steps on the ETH-UCY dataset. As shown in the table, the proposed model outperforms the baseline in the five datasets across both evaluation metrics. Compared to the baseline with the second-best prediction performance, the ADE index increased by 25% to 57%, the FDE index increased by 34% to 62%, and, on average, the ADE and FDE indices increased by 46% and 52%, respectively. These results demonstrate that the proposed model has a strong ability to predict the endpoint of trajectories, with the FDE index improvement being more pronounced than that of the ADE index.

Social-LSTM performed the worst among the 15 baseline models, while SGAN, which optimizes the social pool based on Social-LSTM, significantly improved the prediction performance. None of the baseline models outperformed the proposed model in all indicators on the five datasets. This indicates that the adaptability of the baseline models is not strong, underscoring the strength of the proposed model’s prediction performance.

It is worth noting that TPNet showed the best prediction performance on the HOTEL, UNIV, and ZARA2 datasets among the baseline models, but none of its average indicators were superior. This was due to its poor performance on the ETH dataset, which brought down its average prediction performance. Additionally, the average ADE index of the baseline models was mostly around 0.4 without breaking through, whereas the proposed model achieved a lower ADE index of 0.22, demonstrating a breakthrough in prediction performance.

Most existing models directly mine data from the original trajectory data. In contrast, our model first elevates the dimensionality of the trajectory data by mapping the original trajectory data into a high-dimensional space for exploration. This leads to better trajectory prediction performance compared to baseline models. Additionally, we designed the novel DAGConv module to extract trajectory features at different levels, which is applied in the goal aggregation and CVAE modules, enhancing the predictive performance. Furthermore, unlike previous models, we also considered the similarity between different object motion trajectories and designed the goal feedback module to capture similar trajectory features. This allows for improved trajectory prediction when similar trajectories exist.

Table 2 presents the comparative experimental results of the proposed TFBNet model and the baseline model on the ETH-UCY dataset to predict the next eight time steps. The results indicate that the proposed model outperforms the baseline in both evaluation indicators on all five datasets, with an average ADE increase of 44% and an FDE increase of 60% compared to the second-best model. The improvement in performance is slightly more significant than that of the next 12 steps, further highlighting the high prediction performance and strong adaptability of the proposed model.

**Figure 3:**
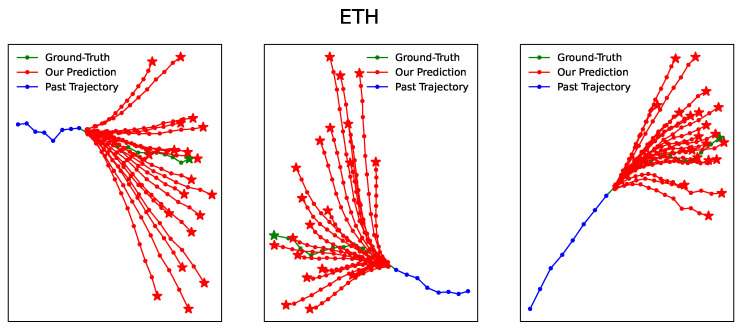
TFBNet’s prediction results on ETH.

**Figure 4:**
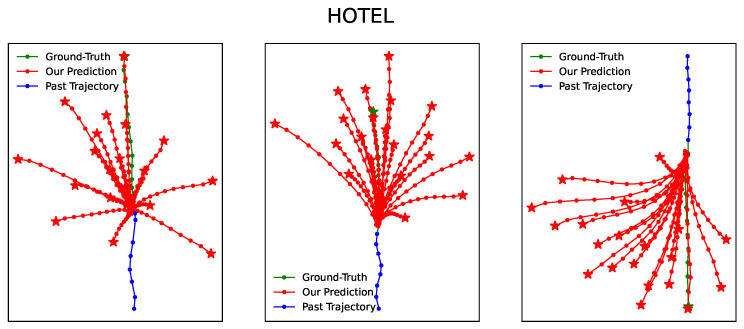
TFBNet’s prediction results on HOTEL.

**Figure 5:**
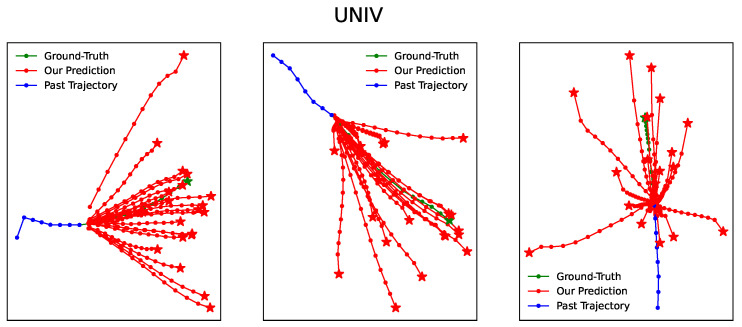
TFBNet’s prediction results on UNIV.

**Figure 6:**
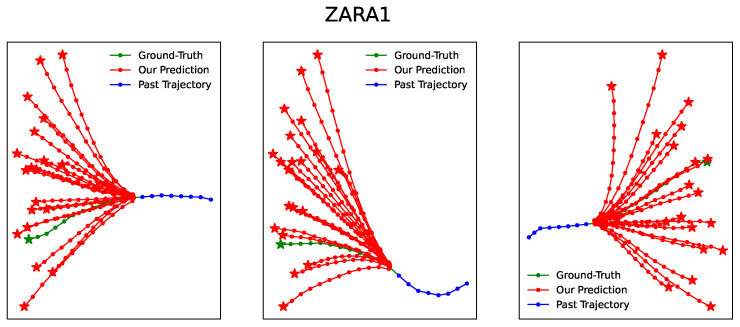
TFBNet’s prediction results on ZARA1.

**Figure 7:**
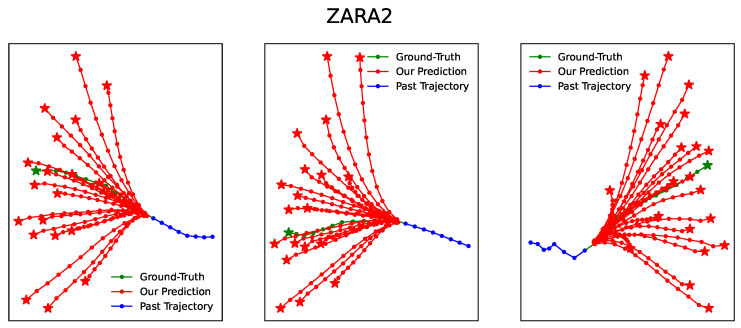
TFBNet’s prediction results on ZARA2.

Figure 3, Figure 4, Figure 5, Figure 6 and Figure 7 illustrate the prediction process of TFBNet, where K = 20. The blue line represents the observed track in the past, the green line represents the actual future track, and the red line represents the future track predicted by the model. The predicted trajectory of TFBNet shows relatively accurate final destinations and relatively divergent predicted future trajectories, which reflect the characteristics of multi-modal prediction well. This again demonstrates the good prediction performance of TFBNet.

To account for the uncertainty in an object’s future trajectory, this paper employs multiple trajectory prediction to simulate the diversity of potential outcomes. Following previous research, K is set to 20 samples, which equates to predicting the future 20 trajectories of the object. Notably, the choice of K can significantly affect the prediction results. To demonstrate this effect on the proposed model’s predictive performance, we conducted further experiments using different K values and prediction lengths ($T_{pred}=8$ and $T_{pred}=12$) and performed a comparative analysis. The results are presented in Table 3 and Table 4. Interestingly, as K increases, the two metrics’ values of the experimental results for all five datasets decrease. This suggests that the proposed model’s predictive performance improves as K increases.

It is worth noting that the current research employs a verification method that compares the best prediction result of multiple trajectories with the actual trajectories. In theory, the more predicted trajectories, the better the prediction performance.

### 4.4. Ablation Experiments

To validate the effectiveness of each module in TFBNet, we conducted an ablation experiment on the ETH-UCY dataset, the results of which are presented in Table 5. The best result is in bold for each dataset. Specifically, we evaluated the performance of TFBNet with individual modules removed: TFBNet-DAGConv with the DAGConv structure removed, TFBNet-MR with the multi-recurrent goal mining module removed, TFBNet-GA with the goal aggregation module removed, and TFBNet-GF with the goal feedback module removed.

The experimental results demonstrate that each module proposed in this paper can improve the model’s predictive performance, with the DAGConv structure having the greatest impact on model performance. This suggests that the DAGConv structure is highly effective in improving the model’s predictive performance. The TFBNet-GA and TFBNet-MR models exhibit similar predictive performance, indicating that the goal aggregation and multi-recurrent goal mining modules have a similar degree of impact on the model. Notably, while the impact of each module on the average displacement error (ADE) is relatively low, their impact on the final displacement error (FDE) is relatively high. This suggests that the models proposed in this paper are especially useful in improving the model’s predictive performance for the end of the trajectory.

## 5. Discussion

A large number of experimental results conducted on five real pedestrian trajectory datasets show that the model proposed in this paper outperforms the baseline model in all indicators. This paper also conducted ablation experiments to verify the effectiveness of each module of the proposed model. At the same time, this paper also conducted analysis experiments on the key parameter K, and the experimental results also indicated that our proposed model has good robustness. Therefore, this paper proposes a model that can effectively predict pedestrian trajectories.

## 6. Conclusions

In this paper, we proposed a novel trajectory prediction network called the trajectory feature-boosting network (TFBNet). By mapping original trajectory data to high-dimensional space and learning the change rule of the trajectory, we provide a new approach for predicting time-series data, such as trajectories. To achieve this, we designed several modules, including a trajectory feature-boosting module, a conditional variational autoencoder (CVAE) module, a multi-recurrent goal mining module, a goal aggregation module, and a goal feedback module. We also introduced a dilated attention gating convolution (DAGConv) structure, which we applied in the CVAE module and goal aggregation module. Our experimental results show that DAGConv can improve the model’s data mining ability and significantly enhance its prediction performance. Our experiments, conducted on five real datasets, demonstrate that the proposed model’s prediction performance is significantly better than that of state-of-the-art methods.

In conclusion, our proposed TFBNet provides a promising approach for predicting time-series data, such as trajectories. Our findings also suggest that the individual modules we designed, including DAGConv, can contribute to enhancing the predictive performance of the model. Overall, our proposed model outperforms state-of-the-art methods, highlighting the potential of our approach in real-world applications.

## Figures and Tables

**Table 1:** Performance comparison of different models for trajectory prediction. K = 20 samples, $T_{pred}$ = 12. The best result is in bold, the second-best result is underlined, and “IMP” represents the relative improvement of the model performance proposed in this paper to the second-best result. ↓ means lower is better. The same for other tables.

Method	ADE/FDE (4.8s) ↓ (m), Best of 20					
	ETH	HOTEL	UNIV	ZARA1	ZARA2	Average
Social-LSTM	1.09/2.35	0.79/1.76	0.67/1.40	0.47/1.00	0.56/1.17	0.72/1.54
SGAN	0.81/1.52	0.72/1.61	0.60/1.26	0.34/0.69	0.42/0.84	0.58/1.18
Sophie	0.70/1.43	0.76/1.67	0.54/1.24	0.30/0.63	0.38/0.78	0.54/1.15
Social-bigat	0.69/1/29	0.49/1.01	0.55/1.32	0.30/0.62	0.36/0.75	0.48/1.00
RSBG	0.80/1.53	0.33/0.64	0.59/1.25	0.40/0.86	0.30/0.65	0.48/0.99
MATF GAN	1.01/1.75	0.43/0.80	0.44 0.91	0.26/0.45	0.26/0.57	0.48/0.90
PSA-GRU	0.79/1.63	0.52/1.07	0.53/1.13	0.41/0.77	0.34/0.74	0.52/1.07
Social-STGCNN	0.64/1.11	0.49/0.85	0.44/0.79	0.34/0.53	0.30/0.48	0.44/0.75
CGNS	0.62/1.40	0.70/0.93	0.48/1.22	0.32/0.59	0.35/0.71	0.49/0.97
PIF	0.73/1.65	0.30/0.59	0.60/1.27	0.38/0.81	0.31/0.68	0.46/1.00
NMMP	0.61/1.08	0.33/0.63	0.52/1.11	0.32/0.66	0.43/0.85	0.41/0.82
FvTraj	0.56/1.14	0.28/0.55	0.52/1.12	0.37/0.78	0.32/0.68	0.41/0.85
DSCMP	0.66/1.21	0.27/0.46	0.50/1.07	0.33/0.68	0.28/0.60	0.41/0.80
STGAT	0.65/1.12	0.35/0.66	0.52/1.10	0.34/0.69	0.29/0.60	0.43/0.83
TPNet	0.84/1.73	0.24/0.46	0.42/0.94	0.33/0.75	0.26/0.60	0.42/0.90
TFBNet (ours)	**0.42/0.58**	**0.15/0.25**	**0.29/0.52**	**0.15/0.25**	**0.11/0.18**	**0.22/0.36**
IMP (%)	25%/46%	37%/45%	30%/34%	42%/44%	57%/62%	46%/52%

**Table 2:** Performance comparison of different models for trajectory prediction. $T_{pred}$ = 8.

Method	ADE/FDE (3.2s) ↓ (m), Best of 20					
	ETH	HOTEL	UNIV	ZARA1	ZARA2	Average
Social-LSTM	0.73/1.48	0.49/1.01	0.41/0.84	0.27/0.56	0.33/0.70	0.45/0.91
SGAN	0.61/1.22	0.48/0.95	0.36/0.75	0.21/0.42	0.27/0.54	0.39/0.78
PSA-GRU	0.58/1.17	0.44/0.87	0.33/0.69	0.25/0.40	0.22/0.46	0.36/0.72
STGAT	0.56/1.10	0.27/0.50	0.32/0.66	0.21/0.42	0.20/0.40	0.31/0.62
TPNet	0.54/1.12	0.19/0.37	0.24/0.53	0.19/0.41	0.16/0.36	0.27/0.56
TFBNet (ours)	**0.29/0.38**	**0.10/0.15**	**0.19/0.32**	**0.09/0.15**	**0.07/0.11**	**0.15/0.22**
IMP	46%/65%	47%/59%	20%/39%	52%/62%	56%/69%	44%/60%

**Table 3:** Experimental results of different K values, $T_{pred}=8$.

Metric	Dataset	K = 1	K = 5	K = 10	K = 15	K = 20	K = 160
ADE	ETH	0.58	0.38	0.33	0.32	0.29	0.20
	HOTEL	0.25	0.17	0.15	0.12	0.10	0.07
	UNIV	0.42	0.27	0.24	0.21	0.18	0.13
	ZARA1	0.24	0.16	0.12	0.12	0.09	0.07
	ZARA2	0.16	0.11	0.09	0.08	0.07	0.05
FDE	**Dataset**	**K = 1**	**K = 5**	**K = 10**	**K = 15**	**K = 20**	**K = 160**
	ETH	1.01	0.63	0.50	0.49	0.38	0.23
	HOTEL	0.45	0.30	0.25	0.18	0.15	0.07
	UNIV	0.79	0.49	0.43	0.38	0.32	0.18
	ZARA1	0.48	0.29	0.21	0.20	0.15	0.09
	ZARA2	0.33	0.21	0.15	0.13	0.11	0.07

**Table 4:** Experimental results of different K values, $T_{pred}=12$.

Metric	Dataset	K = 1	K = 5	K = 10	K = 15	K = 20	K = 160
ADE	ETH	0.85	0.58	0.49	0.47	0.42	0.31
	HOTEL	0.39	0.27	0.23	0.18	0.15	0.11
	UNIV	0.64	0.42	0.37	0.33	0.29	0.21
	ZARA1	0.39	0.25	0.2	0.19	0.15	0.12
	ZARA2	0.27	0.18	0.14	0.13	0.11	0.09
FDE	**Dataset**	**K = 1**	**K = 5**	**K = 10**	**K = 15**	**K = 20**	**K = 160**
	ETH	1.62	1.05	0.81	0.75	0.58	0.38
	HOTEL	0.87	0.57	0.49	0.33	0.25	0.14
	UNIV	1.25	0.8	0.71	0.62	0.52	0.29
	ZARA1	0.82	0.47	0.36	0.33	0.25	0.14
	ZARA2	0.58	0.34	0.26	0.23	0.18	0.11

**Table 5:** Ablation experiments results.

Method	ETH	HOTEL	UNIV	ZARA1	ZARA2	Average
TFBNet-DAGConv	0.49/0.80	0.19/0.36	0.31/0.57	0.18/0.31	0.13/0.24	0.26/0.46
TFBNet-GA	0.43/0.64	0.19/0.36	0.34/0.60	0.16/0.27	0.13/0.23	0.25/0.42
TFBNet-MR	0.46/0.71	0.19/0.37	0.31/0.57	0.18/0.31	0.11/0.18	0.25/0.43
TFBNet-GF	0.43/0.67	0.18/0.36	0.31/0.56	0.16/0.26	0.11/0.19	0.24/0.41
TFBNet	**0.42/0.58**	**0.15/0.25**	**0.29/0.52**	**0.15/0.25**	**0.11/0.18**	**0.22/0.36**

## Data Availability

The dataset used in this paper is a publicly available real trajectory dataset, and the dataset link is as follows: ETH dataset: https://icu.ee.ethz.ch/research/datsets.html (accessed on 5 January 2022). UCY dataset: https://graphics.cs.ucy.ac.cy/research/downloads/crowd-data (accessed on 5 January 2022).
